# PV Panel Model Parameter Estimation by Using Neural Network

**DOI:** 10.3390/s23073657

**Published:** 2023-03-31

**Authors:** Wai Lun Lo, Henry Shu Hung Chung, Richard Tai Chiu Hsung, Hong Fu, Tak Wai Shen

**Affiliations:** 1 Department of Computer Science, Hong Kong Chu Hai College, 80 Castle Peak Road, Castle Peak Bay, Tuen Mun, N.T. Hong Kong, Hong Kong; 2 Department of Electrical Engineering, City University of Hong Kong, Hong Kong; 3 Department of Mathematics and Information Technology, The Education University of Hong Kong, Hong Kong

**Keywords:** model parameters estimation, neural network, photovoltaic panel, maximum power point

## Abstract

Photovoltaic (PV) panels have been widely used as one of the solutions for green energy sources. Performance monitoring, fault diagnosis, and Control of Operation at Maximum Power Point (MPP) of PV panels became one of the popular research topics in the past. Model parameters could reflect the health conditions of a PV panel, and model parameter estimation can be applied to PV panel fault diagnosis. In this paper, we will propose a new algorithm for PV panel model parameters estimation by using a Neural Network (ANN) with a Numerical Current Prediction (NCP) layer. Output voltage and current signals (VI) after load perturbation are observed. An ANN is trained to estimate the PV panel model parameters, which is then fined tuned by the NCP to improve the accuracy to about 6%. During the testing stage, VI signals are input into the proposed ANN-NCP system. PV panel model parameters can then be estimated by the proposed algorithms, and the estimated model parameters can be then used for fault detection, health monitoring, and tracking operating points for MPP conditions.

## 1. Introduction

Solar energy is one of the most popular renewable energy sources as it is pollution free and easy to use. The use of PV panel systems has become popular in the past decade, and research has been done in this area. In [1,2,3], new methods have been proposed to optimize solar energy utilization for maximum power output. However, as PV panels are usually operated without a supervisory mechanism [4], many external or internal factors, such as extreme weather or human faults, may occur, which will degrade the system’s efficiency or even lead to system malfunction or fault. Model parameter estimation for fault detection or health monitoring of PV panels becomes very important to ensure the system’s functionality and efficiency [5].

PV panel defects detection and health monitoring were one of the popular research topics in the past. PV panel monitoring methods include the use of computer vision and machine learning methods [6]. By collecting the image data of the surface conditions of the PV panels, defects and faults can be detected by computer vision methods. These approaches involve the use of machine learning and artificial intelligence for the detection of defects and faults in PV panels. Computer vision methods have the advantages of low cost, non-destructive, and convenience. Recent research also makes use of the self-navigating drone to fly over the PV panels for automatic video data collection [6].

The other performance monitoring approaches include monitoring the PV panel output voltage, current, and power [7,8,9]. The equivalent PV panel circuit model parameters can then be estimated, from which the internal states and health conditions of the PV panels can be identified. In [10,11,12], new approaches have been proposed for the design of the PV panel systems for different loading and operating conditions. In [13,14], new methods have been proposed to monitor the MPP operating conditions of PV panels, and the papers in [15,16,17,18,19,20] proposed new methods for PV panel modeling. The presence of junction capacitance [15] in the PV panel could make a significant deviation between the static I-V characteristics and the dynamic IV characteristics. These discrepancies will increase with sweeping frequencies due to the AC current of the junction capacitance. Methods [16,17,18] have been proposed to obtain a high-precision static I-V model in which load shedding and offline measurement are required. In order to increase the accuracy of PV panel intrinsic parameters estimation, we should take the effect of the junction capacitance [21,22] into account during the sweeping process. 

PV panel intrinsic parameters estimation by I-V dynamics monitoring [7,8,9] is a challenging problem as signals’ derivatives are not available, and we can only monitor the output terminal voltage and current. The mapping between I-V dynamics and intrinsic model parameters could be non-linear and complex due to different environmental conditions. Therefore, computation intelligence [23,24,25] has been used to solve this complex mapping problem. For optimal performance, the maximum power point (MPP) of the PV panels [26,27] can be tracked by using the computation intelligence methods [28,29,30,31,32]. The perturb and observe method [33,34] and the incremental conductance method [35,36] have been proposed to track the MPP of a PV panel. Particle Swarm Optimization (PSO) [25] has been used to estimate the model parameters without taking the effect of the junction capacitance into account. A fault diagnosis device has been developed in [30,31,32] to estimate the model parameter during the MPP locating process. The method in [37] monitors the dynamic I-V characteristics of PV panels, and it provides a non-intrusive method without the frequencies sweeping action. 

It has been shown in [38] that the temperature and irradiance can be used as input parameters for an ANN for the estimation of PV panel output current and voltage. The Gaussian kernel function (GKF) has been introduced into the fuzzy C means (FCM) algorithm [39] for a probabilistic neural network fault diagnosis model. The output of this method can be used for fault class identification. A comprehensive review on the modeling of solar energy systems using ANN is given in [40]. In [41], the inputs of ANN consist of two meteorological variables, temperature and solar irradiance, while the two neurons’ outputs represent the output current and voltage. The work in [42] presents an ANN solution to predict the power generated by the photovoltaic system based on solar radiation measurements. A hybrid evolutionary optimization algorithm [43] has been presented for training an ANN to forecast the energy production of PV panels. In [44], the ANN models with temperature and solar irradiance as inputs are compared with the regression models. A new configurable IoT Open-Source hardware and software I-V curve tracer for PV generators is presented in [45]. 

In the paper [46], a deterministic forecasting model for PV power based on the wavelet transform and deep convolutional neural network was proposed. Liu [47] proposed an output power forecasting method for PV systems by using the ANN back propagation method. In [48], Bonnano proposed a novel ANN-based technique for the modeling of solar cells’ electrical output characteristics. The radiation and voltage are used as input for the ANN, and the outputs are the output current and power. Mellita [49] proposed a 4-layer feed-forward network with solar radiation, ambient temperature, and humidity as input and energy provided by a PV-generator as output. In [50,51], ANN-based models are proposed for the estimation of PV power output.

In [52,53,54,55], new approaches have been developed for PV panel fault diagnosis in which current and voltage data are sampled and model parameters are estimated. This method can provide information about the health conditions of the PV panel and the drift of the model parameters. Experimental studies have been done, and the results show that faulty or damaged panels will give parameters with significant deviation from nominal values. Furthermore, in [53], an online diagnosis method has been developed to monitor the health conditions of two-series-connected PV panels. This approach perturbs the PV panels’ output terminals with a switched inductor circuit, and the perturbation signals of current-voltage characteristics are obtained. Intrinsic model parameters are then estimated by an evolutionary algorithm. The method can monitor the panel health conditions for a long time and extract intrinsic parameters. Experimental studies show that the estimated parameters can reflect the health conditions of the PV panel. 

In [54], a diagnostic method has been developed for monitoring the conditions of two series-connected PV panels. The PV panels are perturbated by a switched-inductor circuit, and the I-V dynamics are monitored by a data acquisition module. A Real-coded Jumping Gene Genetic Algorithm (RJGGA) is proposed to estimate the PV panel model parameters for health monitoring. In [55], the modeling method of the PV panel power output at the reference state has been investigated; this method does not require information on the external factors and uses the regression models obtained by the Least Absolute Shrinkage and Selection Operator (LASSO).

The approaches in [52,53,54,55] involve the use of evolutionary computation (EC) algorithms (e.g., PSO) in which the calculation of objective function values is needed for a number of iterations (or generations) for a population of chromosomes. These EC approaches require significant computation storage and power for implementation. However, in this paper, we will propose a PV panel model parameter estimation method by using a modified ANN; the output voltage and current signals are monitored from which the circuit’s model parameters can be estimated. The VI modeling method has been widely used in automatic PV panels’ defects detection because of its convenience, real-time, and low cost. The capture VI data is used to estimate the PV panel model parameters. The non-linear mapping of operating conditions to model parameters is curve fitted by the ANN, and the final estimated model parameters are fine-tuned by the numerical current predictor (NCP). According to the estimated PV panel model parameters, the health conditions of the PV panel can be evaluated. Furthermore, the conditions for the Maximum Power Point (MPP) of the PV panel can be determined, and the controller can be designed for optimal output performance.

## 2. Methodology

### 2.1. Modeling of PV Panel

The following figure shows the circuit model for the electrical characteristics of the solar cell [15,37]. 

**Figure 1:**
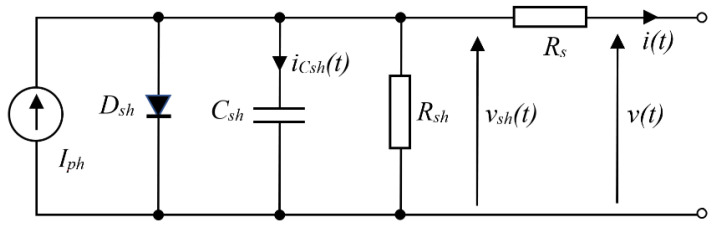
Equivalent circuit (or single-diode model) of a PV panel, I_ph_ is the current source determined by incident light, D_sh_, C_sh_ and R_sh_ are model the p-n junction by diode, capacitor & resistor, R_s_ is series resistance.

According to [15,16], solar panels can be modeled by the electrical model in Figure 1, but parameters should be modified by considering the number of cells connected in series and/or parallel. The model equations in [37] can describe the output characteristics of a PV model. Assume that the parasitic capacitance *C_g_* and resistance *R_g_* between the frame of the panel and the terminals can be neglected (e.g., <1 nF and >10 MΩ). Diode current *i_D_* is given by:

(1) $$i_{D}\left(v_{sh}\right)=I_{o}\left(e^{\frac{v_{sh}}{v_{T}}}-1\right)$$

where *I_o_* is the reverse saturation current, and *v_T_ = n_id_ kT/q*, in which *n_id_* is the ideality factor, *q* is the elementary charge, *k* is the Boltzmann constant, and *T* is the temperature of the *p-n* junction in Kelvin. In Equations (2) and (3), *v* is the panel voltage. The current through *C_sh_* and the rate of change of *v_sh_* can be calculated by Equations (2) and (3). Equation (4) is an implicit equation which is of the utmost importance for computing the current characteristics of the model.

(2) $$i_{Csh}\left(v_{sh},v\right)=I_{ph}-i_{D}\left(v_{sh}\right)-\frac{v_{sh}}{R_{sh}}-\frac{v_{sh}-v}{R_{s}}$$

(3) $$\frac{dv_{sh}}{dt}\left(v_{sh},v\right)=\frac{1}{C_{sh}}\left[I_{ph}-i_{D}\left(v_{sh}\right)-\frac{v_{sh}}{R_{sh}}-\frac{v_{sh}-v}{R_{s}}\right]$$

(4) $$I=I_{ph}-I_{o}\left(e^{\frac{V+IR_{s}}{v_{T}}}-1\right)-\frac{V+IR_{s}}{R_{sh}}$$

### 2.2. PV Panel Output Current Prediction 

The time series of the panel voltage vector **V** and panel current vector **I** contain *N* samples. Let

**V** =     [*v*[0], *v*[1], … *v*[*k*], … *v*[*N*]]     Voltage *v*[*k*] time series
**I** =      [*i*[0], *i*[1], … *i*[*k*], … *i*[*N*]]      Current *i*[*k*] time series
**V_sh_** = [*v_sh_*[0], *v_sh_*[1], … *v_sh_*[*k*], … *v_sh_*[*N*]]  Voltage time series *v_sh_*[*k*] across *C_sh_*
**I_p_** =    [*i_p_*[0], *i_p_*[1], … *i_p_*[*k*], … *i_p_*[*N*]]     Predicted panel current *i_p_*[*k*]

**Figure 2:**
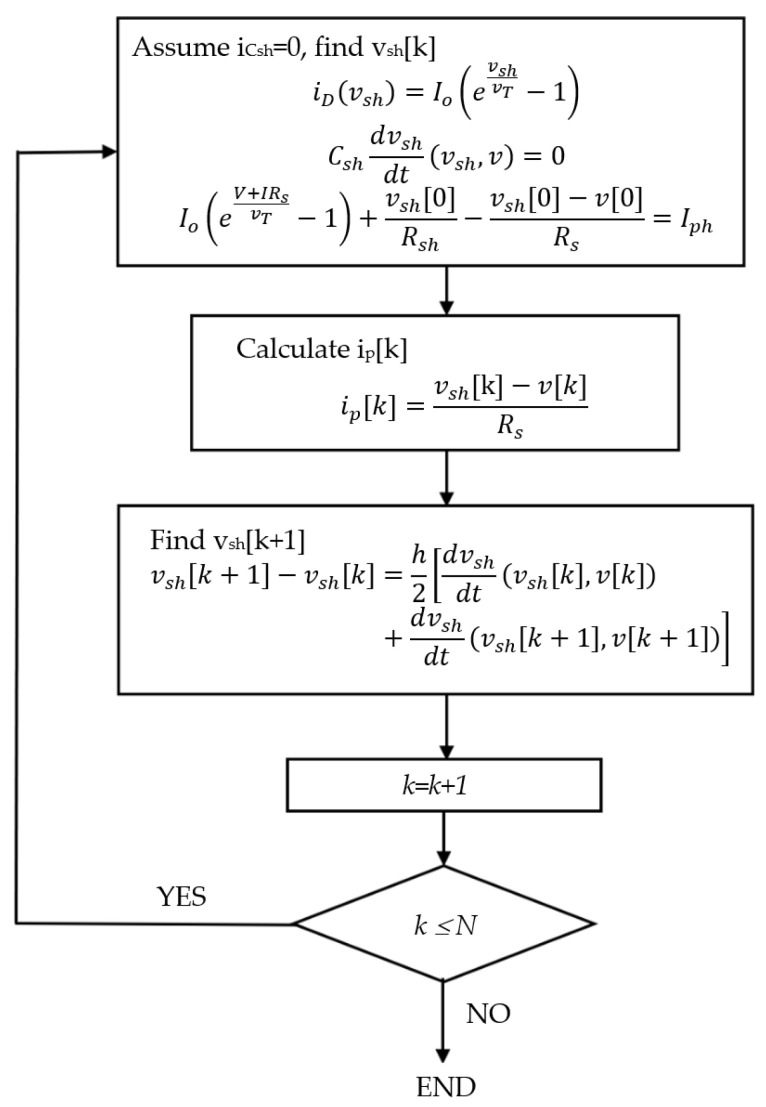
Current Predictor Flow Chart.

The steps of determining *i_p_*[*k*] are listed as follows. The pseudo-code and the flowchart of the current predictor are shown in Algorithm 1 and Figure 2, respectively.

Step 1: The current through the capacitor *C_sh_* is assumed to be zero. *v_sh_*[0] is determined by using (1) and (3). Thus

(5) $$C_{sh}\frac{dv_{sh}}{dt}\left(v_{sh},v\right)=0$$

(6) $$I_{o}\left(e^{\frac{v_{sh}\left[0\right]}{v_{T}}}-1\right)+\frac{v_{sh}\left[0\right]}{R_{sh}}-\frac{v_{sh}\left[0\right]-v\left[0\right]}{R_{s}}=I_{ph}$$

Step 2: *i_p_*[*k*] is calculated by

(7) $$i_{p}\left[k\right]=\frac{v_{sh}\left[k\right]-v\left[k\right]}{R_{s}}$$

Step 3: *v_sh_*[*k +* 1] is obtained by solving a trapezoidal equation with Newton’s method

(8) $$v_{sh}\left[k+1\right]-v_{sh}\left[k\right]=\frac{h}{2}\left[\frac{dv_{sh}}{dt}\left(v_{sh}\left[k\right],v\left[k\right]\right)+\frac{dv_{sh}}{dt}\left(v_{sh}\left[k+1\right],v\left[k+1\right]\right)\right]$$

where *h* is the sampling time interval. The derivative functions on the right-hand side of (8) are obtained by using (3) in the discrete form.

Step 4: *k* is increased by 1.
Step 5: Steps 2 to 4 are repeated until *k = N*.

**Algorithm 1** Current Predictor Pseudo-Code1$\;I_{C_{sh0}}=0$2  While $|I_{C_{sh}}\left[0\right]-I_{C_{sh0}}|>\delta$3 $\;\;\;\;\;V_{sh}\left[0\right]=V_{sh}\left[0\right]+\Delta v$4 $\;\;\;\;\;I_{D}\left[0\right]=I_{o}\left(e^{\frac{V_{sh}\left[0\right]}{V_{T}}}-1\right)$5 $\;\;\;I_{Csh}\left[0\right]=I_{ph}-I_{D}\left[0\right]-\frac{V_{sh}\left[0\right]}{R_{sh}}-\frac{V_{sh}\left[0\right]-V\left[0\right]}{R_{s}}$6 end;7 for *i = 1:i_max_-1*8 $\;\;t\left[i\right]=i\Delta t$9 $\;\;V\left[i\right]=V_{dc}+A_{m}\sin\left[\omega i\Delta t\right]$10$\;\;\;I_{D}\left[i\right]=I_{o}\left(e^{\frac{V_{sh}\left[i\right]}{V_{T}}}-1\right)$11$\;\;\;I_{Csh}\left[i\right]=I_{ph}-I_{D}\left[i\right]-\frac{V_{sh}\left[i\right]}{R_{sh}}-\frac{V_{sh}\left[i\right]-V\left[i\right]}{R_{s}}$12$\;\;\;\frac{dV_{sh}\left[i\right]}{dt}=\frac{I_{Csh}\left[i\right]}{C_{sh}}$13$\;\;\;V_{sh}\left[i+1\right]=V_{sh}\left[i\right]+\Delta t\frac{dV_{sh}\left[i\right]}{dt}$14 $\;\;V\left[i+1\right]=V_{in}\left[i+1\right]$15$\;\;\;I_{p}\left[i+1\right]=\frac{V_{sh}\left[i+1\right]-V\left[i+1\right]}{R_{s}}$16 end

**Figure 3:**
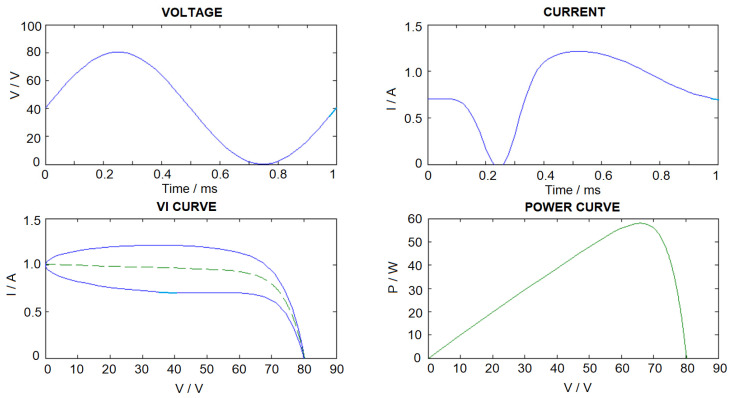
Sample VI and power curve (I_ph_ = 1, I_o_ = 1 × 10^−7^, V_T_ = 5, R_sh_ = 1000, C_sh_ = 1 × 10^−6^, R_s_ = 1). Blue (dynamic characteristics) Green (static characteristics).

For the steady state characteristics of the PV panel, the junction capacitance is not taken into account and steady state output current and the voltage equation is given as follows. The *VI* curve and the power curve are shown in Figure 3. The MPP can be tracked by searching the value of the voltage point for maximum power in Equation (9) or by Equation (10).

(9) $$I_{p}=\frac{R_{sh}}{R_{sh}+R_{s}}\left[I_{ph}-I_{o}\left(e^{\frac{V+IR_{s}}{v_{T}}}-1\right)-\frac{V}{R_{sh}}\right]$$

(10) $$\frac{d}{dV}\left(VI\right)=I+V\frac{dI}{dV}=0\Rightarrow I+V\frac{dI}{dV}\Rightarrow \frac{dI}{dV}=-\frac{I}{V}$$

### 2.3. Proposed Estimation System

**Figure 4:**
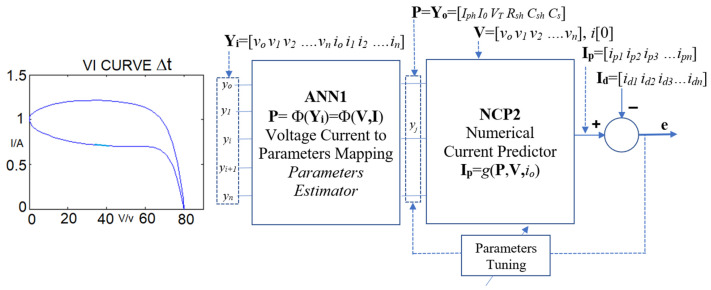
Block Diagrams of the PV panel parameters estimation system.

It is well known that ANN can be used as a universal approximator for complex non-linear functions [56], therefore a PV panel with a given load output perturbation will generate a variation of load current. The structure of the proposed system is given in Figure 4. The VI vector (**Y_i_**) is used as an input-to the ANN1 while the output of ANN1 is the estimated parameter vector (**Y_o_** = **P**); the vectors **Y_i_**, **Y_o_**, and **P** are defined as:

(11) $$Y_{i}=[V\;I]=\;[v[0],\;v[1],\;\ldots \;v[k],\;\ldots \;v[N],\;i[0],\;i[1],\;\ldots \;i[k],\;\ldots \;i[N]]\;Y_{o}=P=[I_{ph}\;I_{o}\;V_{T}\;R_{sh}\;C_{sh}\;R_{s}]$$

For ANN training, normalized estimated parameters (e.g., x/x_max,_) are used in the above vectors. As illustrated in Section 3, a direct approach with only a single ANN1 can only be able to provide accurate estimated parameters with a narrow parameter variation range. Therefore, a second stage NCP2 is connected to the output of ANN1. 

In the proposed structure, a numerical current predictor (NCP) layer is added in the system so as to predict the current **I_p_** according to the estimated model parameters **P** and the current prediction method in Section 2.2. The predicted current vector **I_p_** is compared with the measured current vector **I_d_**, and the estimation error **e** will be used to tune the model parameters vector **P**. Then the ANN1 is backward tuned based on the adjustment of estimated model parameters **P**. The proposed structure is suitable for a system with a fixed known model structure. Therefore, a well-defined numerical model could be designed for the estimation of load current. For the proposed ANN-NCP structure, we apply the VI vectors as inputs for training the ANN1 so as to map the inputs to the output estimated model parameter vectors **P**. After completing the ANN1 training, NCP is connected to the output of ANN1. The NCP will fine-tune the ANN1 so as to match the predicted output current with the exact output current for the whole dataset. 

### 2.4. Estimation of PV Panel Parameters by Using Neural Network

#### 2.4.1. Single Layer ANN Cascade with Current Predictor

**Figure 5:**
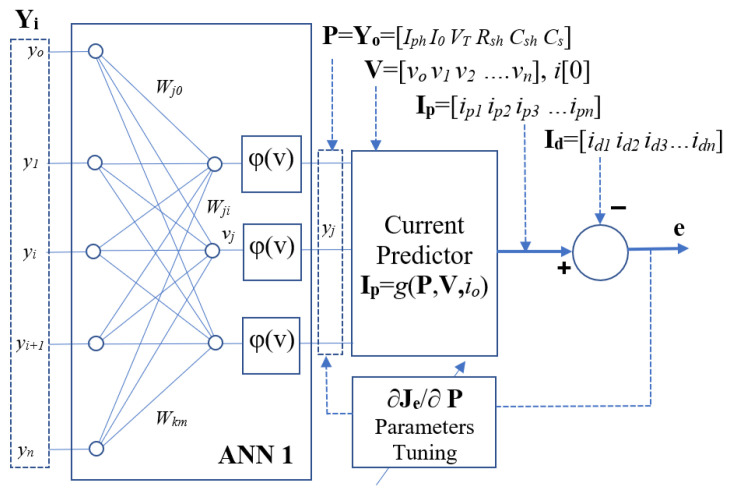
Block Diagrams of the Single-layer ANN1+NCP parameters estimator.

The block diagram for the proposed ANN system is shown in Figure 5. The details of the method are summarized in Appendix A. The ANN1 composes of two layers with activation function *φ*(*v*). The outputs of the ANN1 are the estimated PV panel model parameters **P** = [*I_ph_ I_o_ V_T_ R_sh_ C_sh_ R_s_*]. A PV panel current predictor is connected to the output of the ANN1. 

The numerical current predictor (NCP) can be considered as an extra numerical layer of the Neural Network, the function of the numerical layer is to derive the predicted current vector **I_p_** according to the estimated parameters output **P** of the ANN1, and, therefore, the estimated error (*e*) can be determined. In this paper, the error signal at NCP2 output at iteration *n* is defined as:

(12) $$\begin{array}{c} I_{p}=g\left(P,V,i_{0}\right)\\ e=I_{d}-I_{p} \end{array}$$

where ***P*** = ***Y_o_*** is the output vector of ANN1, which is the (*y_o_/y_omax_*) estimated model parameter vector, *g*(***P***, ***V***, *i*_0_) is the current predictor function of the PV model with parameter vector **P**. **I_d_** is the measured output current vector of the PV panel. 

The predicted current *I_p_*(*t*) (*t = time index, 0 … N_x_*) can be found by using the PV panel current prediction equations in Appendix A. The error cost function ***E***(*n*) at *n* iteration is defined as the sum of square errors between the predicted current vector **I_p_** and the desired (measured) output **I_d_** current vector for *N_x_* data samples.

(13) $$E\left(n\right)=\frac{1}{2}\left[I_{d}-I_{p}\right]{\left[I_{d}-I_{p}\right]}^{T}=\frac{1}{2}ee^{T}$$

The predicted current vector, **I_p_** is a function of estimated model parameter vector, **P** = **Y_o_**, voltage vector **V** and the initial current *i_o_*. For ANN1 with current prediction layer, the gradient of error cost with respect to ANN1 output ∂*E/*∂*y_j_* is a complex function of model parameters **P**.

(14) $$e\left(n\right)=I_{d}\left(n\right)-g(Y_{k}\left(n\right),V,i_{0})\;\;E\left(n\right)=\frac{1}{2}e\left(n\right)e^{T}\left(n\right)$$

(15) $$\frac{\partial E\left(n\right)}{\partial w_{ji}\left(n\right)}=\frac{\partial E\left(n\right)}{\partial y_{j}\left(n\right)}\frac{\partial y_{j}\left(n\right)}{\partial v_{j}\left(n\right)}\frac{\partial v_{j}\left(n\right)}{\partial w_{ji}\left(n\right)}=\frac{\partial E\left(n\right)}{\partial y_{j}\left(n\right)}\varphi^{\prime }\left(v_{j}\left(n\right)\right)y_{i}\left(n\right)\;$$

(16) $$\begin{array}{c} \Rightarrow \frac{\partial E\left(n\right)}{\partial w_{ji}\left(n\right)}=-\delta_{j}\left(n\right)y_{i}\left(n\right)\;\\ \delta_{j}\left(n\right)=-\frac{\partial E\left(n\right)}{\partial v_{j}\left(n\right)}=-\frac{\partial E\left(n\right)}{\partial y_{j}\left(n\right)}\varphi^{\prime }\left(v_{j}\left(n\right)\right) \end{array}$$

The local gradient at neuron *j* is calculated by using the numerical derivative approximation formula Equation (17). The predicted current vector **I_p_**‘ for a small change of model parameter Δ*y_j_* is calculated by current predictor equations. The change of error cost Δ*E(n)* is calculated by Equation (13). Finally, the approximate gradient and the estimated parameter correction are given by:

(17) $$\frac{\partial E\left(n\right)}{\partial y_{j}\left(n\right)}=\frac{\Delta E\left(n\right)}{\Delta y_{j}\left(n\right)}\;\;\Delta y_{j}=-\eta^{\prime }\frac{\Delta E\left(n\right)}{\Delta y_{j}\left(n\right)}$$

The parameter correction is equal to the product of learning rate (*η*’) and error cost gradient. For classical ANN without current predictor, ∂*E/*∂*y_j_* is given by Equation (A11) in Appendix A. However, for ANN1 with current prediction layer, ∂*E/*∂*y_j_* is a complex function of model parameters **P**. Finally, the changes of ANN1 weights are given by:

(18) $$\Delta w_{ji}\left(n\right)=-\eta \frac{\partial E\left(n\right)}{\partial w_{ji}\left(n\right)}=\eta \delta_{j}\left(n\right)y_{i}\left(n\right)$$

The weight correction Δ*w_ji_* is equal to the product of learning rate (*η*), local gradient (*δ*_j_) and the input signal *(y_i_)* of neuron *i.*

#### 2.4.2. Multi-Layer ANN Cascade with Current Predictor

**Figure 6:**
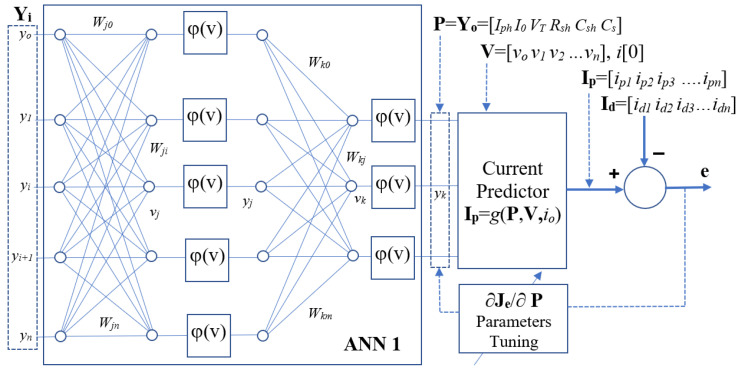
Block Diagrams of the Multi-layer ANN1 parameters estimator.

The structure of the proposed system with multi-layers in ANN1 is shown in Figure 6 The details of the method are summarized in Appendix A. For the output layer in ANN1, the local gradient at the output node *k* is given by:

(19) $$\delta_{k}\left(n\right)=-\frac{\partial E\left(n\right)}{\partial v_{k}\left(n\right)}=-\frac{\partial E\left(n\right)}{\partial y_{k}\left(n\right)}\varphi^{\prime }\left(v_{k}\left(n\right)\right)$$

The above local gradient can be found by using Equation (17). Finally, the parameter corrections and the ANN1 weights’ correction Δ*w_kj_* at output layer are given by:

(20) $$\Delta y_{k}=-\eta^{\prime }\frac{\Delta E\left(n\right)}{\Delta y_{k}\left(n\right)}\;\;\Delta w_{kj}\left(n\right)=\eta \delta_{k}\left(n\right)y_{j}\left(n\right)$$

For the hidden layers in ANN1, the local gradient for the hidden layer neuron *j* is:

(21) $$\delta_{j}\left(n\right)=-\frac{\partial E\left(n\right)}{\partial v_{j}\left(n\right)}=\varphi_{j}^{\prime }\left(v_{j}\left(n\right)\right)\overset{m}{\underset{k=0}{\sum }}\delta_{k}\left(n\right)w_{kj}\left(n\right)$$

(22) $$\Rightarrow \frac{\partial E\left(n\right)}{\partial w_{ji}\left(n\right)}=-\delta_{j}\left(n\right)y_{i}\left(n\right)=-\varphi_{j}\prime \left(v_{j}\left(n\right)\right)y_{i}\left(n\right)\overset{m}{\underset{k=0}{\sum }}\delta_{j}\left(n\right)w_{kj}\left(n\right)\;$$

where *v_j_* is the linear output at hidden neuron *j*. Finally, the changes of ANN weights *w_ji_* are given by:

(23) $$\Delta w_{ji}\left(n\right)=-\eta \frac{\partial E\left(n\right)}{\partial w_{ji}\left(n\right)}=\eta \delta_{j}\left(n\right)y_{i}\left(n\right)$$

The weight correction Δ*w_ji_* is equal to the product of learning rate, local gradient and the input signal of neuron *i*. 

## 3. Results and Analysis

### 3.1. Generating the Training Dataset

**Figure 7:**
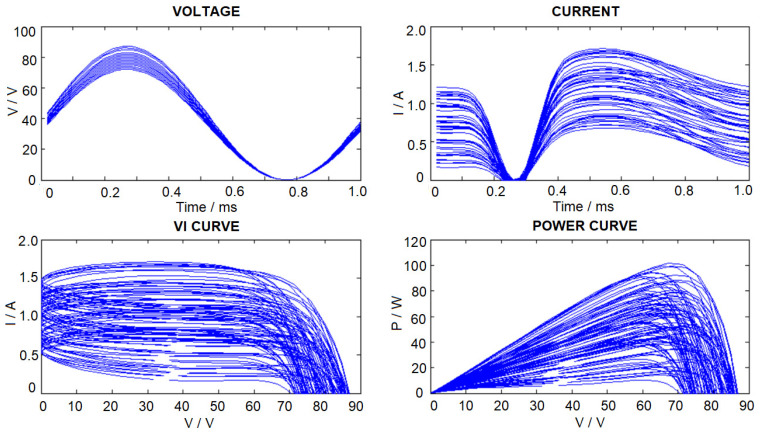
Samples of data curves of the training dataset.

A new method has been proposed [36] to build up the dataset for the ANN training so as to estimate the PV panel model parameters according to the variation of temperature and radiation. PV model parameters have also been estimated based on the steady-state or static voltage-current (VI) characteristics. However, in this paper, the estimated PV panel model parameters are based on the dynamic VI variations. The model parameters include the panel source current determined by incident light, diode parameters, junction capacitance, and resistance, and the panel series resistance. Suppose the PV panel is connected to an electronic load with sinusoidal voltage characteristics, due to the junction capacitance and the non-linear diode characteristics, the resulting PV panel output current will have non-sinusoidal characteristics, as shown in Figure 7. In [33], a method for generating a periodic variation in output voltage by connecting suitable switching devices between two PV panels has been proposed. Therefore, dynamics VI data could be collected through a Digital Acquisition Module installed at the terminal of the PV panels.

In order to evaluate the method in this paper, simulation studies were conducted on servers using 2.6 GHz Intel CPU i7-8700 and 32 GB memory. The PV panel parameter estimation algorithm is implemented in PC by using Python (library used: numpy ver. 1.22.4, pytorch ver. 1.10.1). For generating the ANN training dataset, the PV panel model parameters are allowed to be varied randomly within the maximum and minimum values, as shown in Table 1. As *v_T_* is usually maintained relatively constant for different health conditions [31], *v_T_* is only given ±5% variation. We assume that the PV panel is connected to an electronic load with sinusoidal voltage variation. The sinusoidal load voltage amplitude and dc offset are chosen so as to ensure the variation of output voltage, and current will always be positive and below the practical maximum limits (*V_max_ ≥ V ≥* 0 and *I_max_ ≥ I ≥* 0, 0–3 A, 0–80 V). In this paper, we have used computer simulations to generate a total number of 3693 VI data curves with 50 data samples for each voltage (V) and current vector (I). Samples of training data curves are shown in Figure 7.

### 3.2. ANN + NCP for Parameters Estimation 

**Figure 8:**
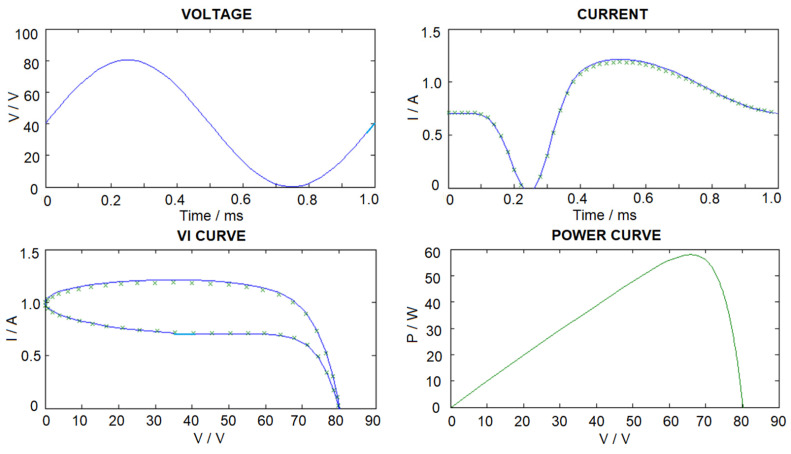
Sample of Actual (solid) and Estimated (x) *V*, *I* and *P* data curves (I_ph_ = 1, I_o_ = 1 × 10^−7^, V_T_ = 5, R_sh_ = 1000, C_sh_ = 1 × 10^−6^, R_s_ = 1). Blue (dynamic characteristics) Green (static characteristics).

The whole generated VI data set is divided into two parts, with 80% of the dataset used as the first part for ANN training, while the second part, with 20% of the dataset, is used for testing and performance evaluation. The estimation errors of 20 data samples and the average estimation error for the whole testing dataset are shown in Table 2. It can be noted that the average estimation error is about 6%. The configurations of the ANN are summarized in Table 2. Figure 8 shows a sample of the actual current and the estimated current. The simulation results of the ANN estimator are summarized in Table 2. 

The dataset consists of 3693 data lines with each data line consists of 50 voltage samples, 50 currents samples in which 2954 data lines are used for training, and 739 data lines are used for testing and performance evaluation. For the ANN1, input dimension 100 (*V*[0] … *V*[N] *I*[0] … *I*[N]) where N = 49, 3 hidden layers, 1st hidden layer 200 nodes, 2nd hidden layer 150 nodes, 3rd hidden layer 50 nodes, output node 6. The ANN learning rate is 0.1. The learning rate of the NCP layer is 0.01.

### 3.3. Performance Evaluation of the Proposed Method

In order to compare the performances of the proposed ANN + NCP method with the traditional approach, a traditional single stage ANN is trained to approximate the non-linear mapping between input VI parameters and the estimated model parameters **P**. The ANN settings of Table 3 are the same as that in Table 2 except the PV model parameters are given a ±90% variation while *v_T_* is given a ±5% variation for generating the data set. The simulation results are summarized in Table 3. It can be noted that the direct ANN method has an average estimation error of 9.58%, while the ANN + NCP give an average estimation error of 7.24% for ±90% variation range. The proposed method has a better performance than the traditional direct ANN approach.

In [37], a method for the fault diagnosis of PV Panels using dynamic I-V characteristics has been proposed. The output current and voltage of the PV panel are sampled by a Fault Diagnosis Device (FDD) during the sweeping process initiated by the MPP tracker. For the MPP tracker, the impedance of an electronic load is controlled by computer which can communicate with the FDD through a power line communication device. A modified Particle Swarm Optimization (PSO) algorithm was developed to estimate the PV panel model parameters. The proposed method can provide estimated model parameters and information for the health conditions of the PV panel. Experimental results also show that model parameters of faulty PV panels have significant deviation from nominal values, therefore, this method can be used for fault diagnosis of PV panels. The proposed method has the advantages of scalability, modularity, and remote-control capability.

Both the proposed method and the method in [37] involve measurement of voltage and current signals. However, during each iteration, PSO approach involves calculations of the predicted current vectors and the objective function values for all chromosomes of the population for a number of generations. The objective function is defined as the sum of square error between the predicted current and the actual measure current. This PSO approach requires significant computation steps and storage. However, in our proposed ANN + NCP method, once the ANN training process is completed, the computation load and storage required is less than that of the PSO approach, which involves evolution of chromosomes in each generation. For each parameter estimation process, the method in [37] needs to manipulate a population of chromosomes. 

In order to further evaluate the performance of the proposed method, the experimental dataset in [37] is used to test the performances of the proposed method. The experimental dataset consists of voltage and current data sequences measured under a load perturbation test for practical PV panels (Sungen SG-NH80-GG 80 W, a-Si type). In the experiment, the panels are connected to an electronic load HP6050A to emulate the sweeping process in the MPP tracker in a PV system. A computer is used to control the impedance of the electronic load and communication with the device through power line communications. The experimental VI data series are input to the trained ANN + NCP system and the estimation results are summarized in Table 4. It can be noted that model parameters estimated by our proposed method are similar to estimated results in [37]. The estimated parameters by our proposed method are consistent with the results in [37], and the average discrepancies is about 8%. 

## 4. Discussion

In [33], a perturb and observe algorithm to track the maximum power point condition of a PV panel is proposed. Starting from an initial estimate of maximum power, the actual PV current and voltage are measured at specific intervals and the power is recorded and compared with the current maximum power. Finally, the MPP operating point of the PV panel is estimated. In [34], a new method for tracking the MPP of a PV panel by using a Cuk converter is proposed in which the Ćuk converter is used as an impedance matching device between input and output by adjusting the duty cycle of the converter circuit. As PV power production is highly dependent on environmental and weather factors (e.g., solar irradiance and ambient temperature etc.), a single control condition cannot ensure the PV panel will achieve optimal performance and efficiency in different operating conditions. Therefore, incremental conductance methods for the MPP tracking of PV panels have been proposed in [35,38]. In these approaches, the voltage and current of the PV module are measured by the MPPT controller; the duty cycle of the converter varies according to different conductance level. The incremental conductance algorithm detects the slope of the P–V curve, and the MPP is tracked by searching the peak of the P–V curve.

The perturb and observe method and the incremental conductance method have many practical applications for tracking the MPP of PV panels in different operating conditions. The proposed method in this paper also involves measuring the output voltage current, but the method is designed to estimate the PV panel model parameters. The MPP operating point can then be estimated by tracking the voltage for maximum power along the VI curve as described by the PV model equation. Furthermore, the estimated model parameters can be used for health monitoring or fault detection of the PV panel. Therefore, the method proposed in this paper not only could provide the model parameters for health monitoring and fault detection, the MPP could also be derived from tracking the VI curve of the estimated PV panel model.

For monitoring the health conditions of PV panels, the value of *I_ph_* cannot be used as the only indicator for health conditions of a PV panel as it is well known that irradiance can affect the *I_ph_* directly. A damaged PV panel will have a smaller value of *I_ph_*. Furthermore, *I_ph_* could also be affected by partial shading. For an open PV farm without partial shading, health conditions of PV panels could be evaluated by observing the estimated *I_ph_*. According to [37], *v_T_* does not have significant variations for PV panel with different health conditions, therefore *v_T_* should not be used as an indicator for evaluating health conditions. According to extensive simulation results, the proposed algorithm has a limitation that the estimation accuracy will decrease when *v*_T_ has a large variation over 10%. However, it has been mentioned in [37] that *v*_T_ is relatively constant in practice. The value of *v_T_* can be further fine-tuned by an evolutionary computation algorithm but these approaches usually need more computation load. An unhealthy PV panel will have a large value of *R_s_* and a smaller value of *C_sh_*. An unhealthy PV panel with physical damage will change the value of *R_s_*. Therefore, by observing the VI characteristics after load changes or injected load perturbation, we can estimate the values of various parameters (e.g., *I_ph_*, *R_s_* and *C_sh_*) and, therefore, we can evaluate the health conditions of PV panels.

## 5. Conclusions

This paper proposed a modified ANN parameters estimator for PV panel model parameter estimation. According to the input VI data vectors for a standard perturbation test, the estimator can give an accuracy of about 6%. The ANN is designed as a VI pattern recognizer to approximate the mapping between VI data patterns and the PV model parameters. The estimated PV model parameters are then passed to the numerical current predictor (NCP). The NCP will fine-tune the PV model parameters estimated by the ANN so as to reduce the prediction current error. The proposed method has better accuracy than the direct single-stage ANN method. The proposed method has been tested by practical experimental dataset. The outputs of the proposed system are the estimated PV model parameters which can be used to evaluate the health conditions of the PV panel. The estimated model parameters can also be used to track the MPP of PV panels and a power converter controller can be designed for optimal PV panel output performance. In conclusion, an automatic PV panels model parameter estimation algorithm was proposed in this paper which can be used for health monitoring, fault detection, and MPP tracking of solar PV panels.

## Figures and Tables

**Table 1:** Estimation ranges of the model parameters. (Solar panel reference model: Sungen SG-NH80-GG 80 W, a-Si type).

Model Parameters	Nominal	Minimum Value	Maximum Value
*I_ph_*	1	0.25	1.75
*I_o_*	1 × 10^−7^	0.25 × 10^−7^	1.75 × 10^−3^
*V_T_*	5	4.75	5.25
*R_sh_*	1000	250	1750
*C_sh_*	1 × 10^−6^	0.25 × 10^−7^	1.75 × 10^−5^
*R_s_*	1	0.25	1.75

**Table 2:** ANN + NCP (Input 100, V[0] … V[49], i[0] … i[49], three hidden layers, 1st hidden layer 200 nodes, 2nd hidden layer 150 nodes, 3rd hidden layer 50, output 6 model parameters values, learning rate 0.1, NPC learning rate 0.01. N = 3693, Training data 2951, Testing data 739). (**a**) Estimation error of the model parameters (first 20 sorted data samples). (**b**) Summary of average estimation error of the model parameters for testing dataset.

(a)												
Model Parameter Values						Parameter Error (%)						Avg P Err
*I_ph_*	*I_o_*	*V_T_*	*R_sh_*	*C_sh_*	*R_s_*	*I_ph_*	*I_o_*	*V_T_*	*R_sh_*	*C_sh_*	*R_s_*	*P%*
1.185652	4.30 × 10^−8^	4.960991	625.8864	1.49 × 10^−6^	1.033527	0.252%	2.046%	0.826%	1.889%	2.895%	0.254%	1.360%
1.599342	1.21 × 10^−7^	5.000439	1080.711	7.45 × 10^−7^	1.159845	0.271%	0.057%	0.096%	1.856%	4.894%	1.099%	1.379%
1.258293	1.00 × 10^−7^	5.008741	482.2779	1.50 × 10^−6^	1.561261	0.863%	0.305%	0.054%	1.766%	1.135%	5.036%	1.527%
0.927406	9.93 × 10^−8^	5.022864	353.1422	7.70 × 10^−7^	1.24505	1.415%	3.360%	0.181%	1.430%	2.071%	0.844%	1.550%
1.348611	1.60 × 10^−7^	4.961369	559.2404	1.58 × 10^−6^	1.225154	2.932%	5.181%	0.065%	0.431%	0.004%	1.274%	1.648%
0.979375	1.34 × 10^−7^	5.011321	377.6653	1.54 × 10^−6^	1.643884	0.216%	1.145%	0.076%	4.982%	0.200%	3.298%	1.653%
1.484035	1.12 × 10^−7^	5.007811	731.1763	1.60 × 10^−6^	1.023995	3.667%	3.824%	0.049%	0.248%	0.061%	2.490%	1.723%
1.020282	1.21 × 10^−7^	4.993472	935.1544	6.19 × 10^−7^	1.016744	3.367%	3.600%	0.102%	1.796%	1.169%	0.516%	1.758%
1.098789	1.39 × 10^−7^	5.008699	907.7543	1.11 × 10^−6^	1.266457	1.481%	1.846%	0.037%	0.648%	3.440%	3.353%	1.801%
0.765435	1.21 × 10^−7^	5.011792	1577.93	1.45 × 10^−6^	1.113147	4.732%	0.591%	0.074%	2.386%	2.943%	0.239%	1.827%
1.093242	6.72 × 10^−8^	5.002325	431.5167	1.11 × 10^−6^	1.601015	1.868%	5.745%	0.443%	0.779%	0.990%	1.250%	1.846%
0.464915	1.31 × 10^−7^	5.007424	363.4642	1.38 × 10^−6^	1.038277	0.166%	5.847%	0.152%	1.301%	1.345%	2.629%	1.907%
1.679152	1.40 × 10^−7^	5.001899	392.504	9.61 × 10^−7^	0.913558	3.592%	3.414%	0.031%	1.480%	2.111%	1.059%	1.948%
1.545015	1.03 × 10^−7^	4.99852	837.1954	1.51 × 10^−6^	1.607093	1.501%	0.386%	0.074%	5.052%	3.338%	1.675%	2.004%
0.652602	1.52 × 10^−7^	4.957675	1126.454	9.85 × 10^−7^	0.988403	2.446%	4.427%	0.570%	0.851%	1.197%	2.745%	2.039%
1.587799	8.89 × 10^−8^	5.028851	714.428	1.60 × 10^−6^	1.696029	0.736%	2.121%	0.480%	4.601%	3.195%	1.148%	2.047%
0.506147	9.19 × 10^−8^	4.999409	1504.394	1.18 × 10^−6^	1.384393	1.435%	2.406%	0.078%	7.721%	0.236%	0.580%	2.076%
1.320259	1.31 × 10^−7^	5.005815	1679.147	6.35 × 10^−7^	1.022127	3.239%	1.304%	0.290%	1.483%	4.505%	1.848%	2.112%
1.057464	7.21 × 10^−8^	5.023832	663.0781	1.65 × 10^−6^	1.226728	0.325%	0.222%	0.396%	6.801%	3.507%	1.554%	2.134%
0.806142	7.84 × 10^−8^	4.982985	1331.262	1.48 × 10^−6^	1.098663	0.716%	4.841%	0.432%	4.059%	2.300%	0.498%	2.141%
(b)												
**Average Parameter Error (%) (N = 739)**												**Avg P Err**
***I_ph_***		***I_o_***		***V_T_***		***R_sh_***		***C_sh_***		***R_s_***		***P%***
5.175%		8.960%		0.448%		8.100%		4.735%		7.982%		5.900%

**Table 3:** ANN + NCP (Input 100, V[0] … V[49], i[0] … i[49], three hidden layers, 1st hidden layer 200 nodes, 2nd hidden layer 150 nodes, 3rd hidden layer 50, output 6 model parameters values, learning rate 0.1, NPC learning rate 0.01. (N = 3693, Training data 2951, Testing data 739). (**a**) Estimation error of the model parameters (20 data samples), Direct ANN method, and ANN + NCP method (grey). (**b**) Summary of average estimation error of the model parameters for testing dataset, direct ANN method, and ANN+NCP method (grey).

(a)												
Model Parameter Values						Parameter Error (%)						Avg P Err
I_ph_	I_o_	V_T_	R_sh_	C_sh_	R_s_	I_ph_	I_o_	V_T_	R_sh_	C_sh_	R_s_	P%
1.630083	1.12 × 10^−7^	4.994128	546.4698	7.65 × 10^−7^	0.984176	6.26%	7.91%	0.17%	4.84%	1.13%	23.18%	7.25%
						2.11%	5.93%	0.06%	4.49%	2.02%	9.63%	4.04%
1.257783	9.99 × 10^−8^	4.961331	527.7401	1.36 × 10^−6^	1.710035	6.99%	27.13%	0.88%	20.06%	2.29%	0.67%	9.67%
						3.23%	1.67%	0.71%	0.38%	3.70%	3.36%	2.17%
1.289115	1.24 × 10^−7^	5.044551	1851.599	5.57 × 10^−7^	1.649876	9.00%	7.83%	0.74%	32.54%	1.23%	4.13%	9.24%
						5.91%	8.00%	0.55%	1.83%	4.80%	7.61%	4.78%
1.199209	9.24 × 10^−8^	4.995275	1529.833	8.60 × 10^−7^	1.4266	4.93%	0.93%	0.16%	19.09%	7.85%	3.96%	6.15%
						2.82%	1.78%	0.26%	1.79%	2.31%	3.38%	2.06%
1.250273	1.80 × 10^−7^	5.005995	1301.456	9.57 × 10^−7^	1.091804	5.60%	10.34%	0.56%	1.55%	12.87%	10.48%	6.90%
						2.17%	1.06%	0.27%	3.89%	2.89%	2.71%	2.17%
1.334862	1.02 × 10^−7^	5.011411	1865.791	8.95 × 10^−7^	1.356343	12.76%	4.28%	0.03%	22.44%	10.06%	1.42%	8.50%
						6.85%	6.04%	0.10%	9.50%	3.76%	4.12%	5.06%
1.390933	7.37 × 10^−8^	5.004601	1561.934	1.56 × 10^−6^	1.385632	5.43%	5.88%	0.13%	12.95%	1.27%	1.57%	4.54%
						6.38%	5.76%	0.02%	3.07%	0.52%	6.76%	3.75%
0.423632	7.22 × 10^−8^	4.998006	1039.917	1.64 × 10^−6^	1.701006	2.55%	4.82%	0.19%	34.30%	0.88%	8.67%	8.57%
						2.26%	1.67%	0.08%	1.17%	1.06%	1.94%	1.36%
0.70081	1.03 × 10^−7^	5.020087	998.6866	1.60 × 10^−6^	0.779306	1.39%	0.07%	0.41%	1.49%	4.32%	10.37%	3.01%
						3.38%	7.06%	0.41%	0.33%	5.77%	6.69%	3.94%
0.947303	1.58 × 10^−7^	4.964277	1710.87	9.05 × 10^−7^	0.963713	6.62%	10.42%	0.45%	21.24%	5.70%	6.04%	8.41%
						4.09%	6.63%	0.47%	6.34%	3.64%	6.26%	4.57%
1.875968	1.85 × 10^−7^	4.972942	1291.076	9.33 × 10^−7^	1.513059	3.80%	7.77%	0.37%	1.03%	5.64%	17.88%	6.08%
						4.85%	4.89%	0.47%	2.87%	3.00%	2.28%	3.06%
1.759556	1.48 × 10^−7^	5.006813	1480.449	1.45 × 10^−6^	0.91177	4.35%	2.92%	0.16%	19.72%	1.91%	1.99%	5.17%
						1.07%	3.03%	0.02%	4.31%	5.49%	9.60%	3.92%
0.686874	1.02 × 10^−7^	4.952369	1412.45	4.85 × 10^−7^	1.74991	2.19%	20.01%	0.97%	16.26%	7.70%	3.82%	8.49%
						1.95%	0.28%	1.33%	4.19%	0.90%	0.11%	1.46%
0.604315	1.78 × 10^−7^	5.004975	518.8658	7.25 × 10^−7^	1.001697	12.09%	7.70%	0.33%	9.53%	3.54%	1.32%	5.75%
						2.04%	9.09%	0.20%	0.27%	9.51%	1.57%	3.78%
0.857808	1.25 × 10^−7^	4.996854	953.0882	1.64 × 10^−6^	0.917745	5.62%	4.70%	0.15%	1.26%	2.83%	1.33%	2.65%
						7.70%	9.75%	0.16%	4.10%	1.83%	2.61%	4.36%
0.910657	1.15 × 10^−7^	5.006177	1419.811	6.77 × 10^−7^	0.878179	4.18%	2.11%	0.14%	6.24%	10.22%	11.68%	5.76%
						2.31%	6.25%	0.27%	4.70%	3.56%	1.10%	3.03%
1.807161	1.62 × 10^−7^	5.027173	1415.727	1.53 × 10^−6^	1.100778	4.63%	8.51%	0.52%	0.12%	0.27%	20.59%	5.77%
						3.35%	1.88%	0.37%	8.54%	1.28%	3.30%	3.12%
1.381415	9.77 × 10^−8^	5.01047	870.6045	1.12 × 10^−6^	1.521624	6.85%	6.24%	0.11%	6.00%	5.39%	5.52%	5.02%
						0.32%	2.82%	0.12%	6.66%	0.07%	2.79%	2.13%
1.084105	6.73 × 10^−8^	5.021533	1725.341	1.01 × 10^−6^	1.223236	6.78%	21.86%	0.42%	27.43%	5.85%	6.63%	11.49%
						5.69%	3.94%	0.52%	3.54%	2.50%	2.69%	3.15%
0.390577	1.57 × 10^−7^	5.005679	1005.25	9.06 × 10^−7^	1.440104	2.20%	3.06%	0.24%	1.54%	1.04%	1.56%	1.61%
						2.96%	4.13%	0.02%	1.05%	8.75%	0.74%	2.94%
(**b**)												
						**Average Parameter Error (%)**						**Avg P Err**
						***I_ph_***	***I_o_***	***V_T_***	***R_sh_***	***C_sh_***	***R_s_***	***P%***
Variation Range (N = 477)						±90%	±90%	±5%	±90%	±90%	±90%	
Direct ANN method						5.93%	15.38%	0.48%	15.12%	5.40%	15.17%	9.58%
ANN + NCP method						6.36%	12.61%	0.47%	10.08%	5.04%	8.90%	7.24%

**Table 4:** Testing of proposed method by experimental data in [37]. (**a**) Summary of Reference parameters in [37] and Estimated Model Parameters by proposed method. Comparisons between estimated model parameters in [37] and the model parameters estimated by proposed method with experimental data in [37] used as input. (Dataset 1) High temperature condition—the lights are turned ON for 2 min and the panels are heated up. (Dataset 2) Low temperature condition—the lights are just turned ON and the panels are still cool. * Avg ΔP% = Average discrepancies between P_ref_ and P. (**b**) Measure VI curve and estimated VI curve by proposed method.

(a)													
Panel	P_ref_ Estimated Parameter Values Reference [37]						P Estimated Parameter Values (Proposed Method)						* Avg
	*I_ph_*	*I_o_*	*V_T_*	*R_sh_*	*C_sh_*	*R_s_*	*I_ph_*	*I_o_*	*V_T_*	*R_sh_*	*C_sh_*	*R_s_*	*ΔP%*
Dataset 1	1.18	4.13 × 10^−6^	6.95	427	3.20 × 10^−7^	1.86	1.256211	3.70 × 10^−6^	7.529572	482.5856	2.86 × 10^−7^	1.748339	9.1%
Dataset 2	1.1	3.14 × 10^−6^	7.25	449	2.93 × 10^−7^	1.74	0.979706	2.82 × 10^−6^	6.924886	433.4232	2.78 × 10^−7^	1.883403	7.0%
(**b**) (Blue-measured results, Green-results by proposed method)													
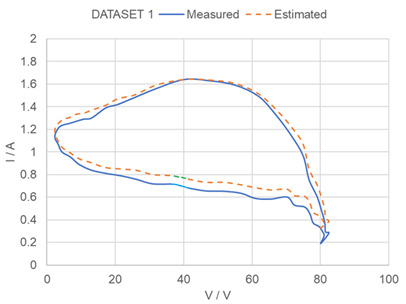							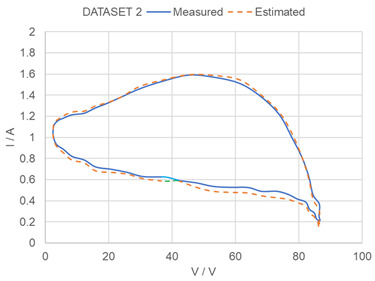						

## Data Availability

Not applicable.

## Appendix A

### Appendix A.1. Single Layer ANN with Current Predictor

The error signal at NCP2 output at iteration *n* is defined as:

(A1) $$I_{p}=g\left(P,V,i_{0}\right)=g\left(Y_{p},V,i_{0}\right)\;E\left(n\right)=\frac{1}{2}\left[I_{d}-I_{p}\right]{\left[I_{d}-I_{p}\right]}^{T}=\frac{1}{2}ee^{T}$$

where **P** = ***Y_o_*** is the output vector of ANN1 in Equation (11) which is the normalized estimated model parameter vector, *g*(***P, V****, i_0_*) is the current predictor function of the PV model with parameter vector **P. I_d_** is the measured output current vector of the PV panel. The output of the ANN1 are the estimated model parameters. The predicted current *I_p_*(*t*) (*t = time index*, 0 *… N_x_*) can be found by using the PV panel current predictor equations:

(A2) $$I_{p}\left(t+1\right)=h\left(P,v_{sh}\left(t\right),v\left(t\right),v\left(t+1\right),\Delta t\right)\;t=0\ldots N_{x}$$

(A3) $$V_{sh}\left(t+1\right)=V_{sh}(t)+\frac{\Delta t}{C_{sh}}I_{sh}(t)$$

(A4) $$I_{p}\left(t+1\right)=\frac{1}{R_{s}}\left(V_{sh}(t+1)-V(t+1)\right)$$

The error cost function ***E***(*n*) in Equation (13) at *n* iteration is defined as the sum of square error between the predicted (**I_p_**) and measured current (**I_d_**) for *N_x_* data samples For a classical neural network, the linear output *v_j_*(*n*) of neuron *j* at iteration *n* is given by:

(A5) $$v_{j}\left(n\right)=\overset{m}{\underset{i=1}{\sum }}w_{ji}\left(n\right)y_{i}\left(n\right)\;y_{j}\left(n\right)=\varphi \left(v_{j}\left(n\right)\right)$$

(A6) $$\frac{\partial E\left(n\right)}{\partial w_{ji}\left(n\right)}=\frac{\partial E\left(n\right)}{\partial v_{j}\left(n\right)}\frac{\partial v_{j}\left(n\right)}{\partial w_{ji}\left(n\right)}$$

(A7) $$\frac{\partial v_{j}\left(n\right)}{\partial w_{ji}\left(n\right)}=y_{i}\left(n\right)$$

For ANN without the current prediction layer $e_{j}\left(n\right)=d_{j}\left(n\right)-y_{j}\left(n\right)$

(A8) $$\begin{array}{c} \frac{\partial E\left(n\right)}{\partial y_{j}\left(n\right)}=-e_{j}\left(n\right)\\ \frac{\partial y_{j}\left(n\right)}{\partial v_{j}\left(n\right)}=\varphi^{\prime }\left(v_{j}\left(n\right)\right) \end{array}$$

(A9) $$\delta_{j}\left(n\right)=-\frac{\partial E\left(n\right)}{\partial v_{j}\left(n\right)}=-\frac{\partial E\left(n\right)}{\partial y_{j}\left(n\right)}\frac{\partial y_{j}\left(n\right)}{\partial v_{j}\left(n\right)}=e_{j}\left(n\right)\varphi \prime \left(v_{j}\left(n\right)\right)$$

(A10) $$\frac{\partial E\left(n\right)}{\partial w_{ji}\left(n\right)}=-\delta_{j}\left(n\right)y_{i}\left(n\right)=-e_{j}\left(n\right)\varphi^{\prime }\left(v_{j}\left(n\right)\right)y_{i}\left(n\right)$$

For classical ANN without current predictor, ∂*E/*∂*y_i_* is given by Equation (A8). However, for ANN1 with current prediction layer, ∂*E/*∂*y_i_* is a complex function of model parameters.

(A11) $$\begin{array}{c} \frac{\partial E\left(n\right)}{\partial w_{ji}\left(n\right)}=\frac{\partial E\left(n\right)}{\partial y_{j}\left(n\right)}\frac{\partial y_{j}\left(n\right)}{\partial v_{j}\left(n\right)}\frac{\partial v_{j}\left(n\right)}{\partial w_{ji}\left(n\right)}\;\\ \frac{\partial E\left(n\right)}{\partial y_{j}\left(n\right)}=-e\left(n\right)\frac{\partial g^{T}\left(n\right)}{\partial y_{j}\left(n\right)} \end{array}$$

(A12) $$\Rightarrow \frac{\partial E\left(n\right)}{\partial w_{ji}\left(n\right)}=-e\left(n\right)\frac{\partial g^{T}\left(n\right)}{\partial y_{j}\left(n\right)}\varphi^{\prime }\left(v_{j}\left(n\right)\right)y_{i}\left(n\right)=-\delta_{j}\left(n\right)y_{i}\left(n\right)$$

(A13) $$\delta_{j}\left(n\right)=-\frac{\partial E\left(n\right)}{\partial y_{j}\left(n\right)}\varphi^{\prime }\left(v_{j}\left(n\right)\right)=e\left(n\right)\frac{\partial g^{T}\left(n\right)}{\partial y_{j}\left(n\right)}\varphi^{\prime }\left(v_{j}\left(n\right)\right)$$

The local gradient at neuron *j* is calculated by using the numerical derivative approximation formula Equation (17). The predicted current vector **I_p_**’ for a small change of model parameter Δ*y_j_* is calculated by current predictor equations. The change of error cost Δ*E(n)* is calculated by Equation (A1). The weight correction Δ*w_ji_* is equal to the product of learning rate (*η*), local gradient (δ*_j_*) and the input signal *(y_i_)* of neuron *i.*

### Appendix A.2. Multi-Layer ANN Cascade with Current Predictor

For the ANN1 output layer, the local gradient at output node *k* is given by:

(A14) $$\delta_{k}\left(n\right)=-\frac{\partial E\left(n\right)}{\partial v_{k}\left(n\right)}=-\frac{\partial E\left(n\right)}{\partial y_{k}\left(n\right)}\varphi^{\prime }\left(v_{k}\left(n\right)\right)=e\left(n\right)\frac{\partial g^{T}\left(n\right)}{\partial y_{k}\left(n\right)}\varphi^{\prime }\left(v_{k}\left(n\right)\right)$$

The local gradient can be found by using Equation (17). For output and hidden layers, the weight correction Δ*w_ki_* and Δ*w_ji_* are equal to the product of learning rate, local gradient and the input signal of neuron.

(A15) $$\Delta w_{kj}\left(n\right)=\eta \delta_{k}\left(n\right)y_{j}\left(n\right)\;\Delta w_{ji}\left(n\right)=\eta \delta_{j}\left(n\right)y_{i}\left(n\right)$$
